# Supplementary material for: Dynamically regulated two-site interaction of viral RNA to capture host translation initiation factor
Source: Nat Commun. 2023 Aug 28;14:4977. doi: 10.1038/s41467-023-40582-6 (PMC10462655; doi:10.1038/s41467-023-40582-6)
Supplement: Supplementary file 1 — Supplementary Information [file 41467_2023_40582_MOESM1_ESM.pdf]

Supplementary Information

Dynamically regulated two-site interaction of viral RNA to capture host translation initiation factor

Shunsuke Imai[1,#,*], Hiroshi Suzuki[2,#], Yoshinori Fujiyoshi[2], and Ichio Shimada[1,3,*]

[1] RIKEN Center for Biosystems Dynamics Research, Tsurumi-ku, Yokohama 230-0045, Japan.
[2] Cellular and Structural Physiology Laboratory (CeSPL), Tokyo Medical and Dental University, Bunkyo-ku, Tokyo 113-8510, Japan.
[3] Graduate School of Integrated Sciences for Life, Hiroshima University, Higashi-Hiroshima 739-8528, Japan

# These authors contributed equally
* Corresponding authors

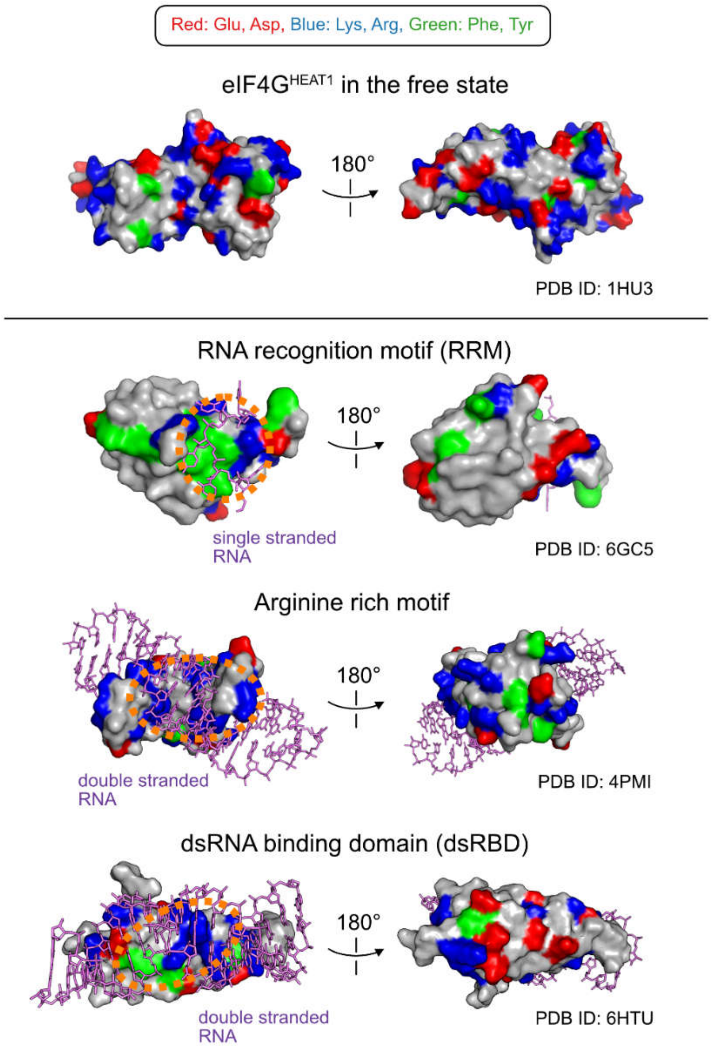

Supplementary Fig. 1 | Comparison of the surface residue distributions of eIF4G^{HEAT1} and typical RNA binding proteins

Surface representations of the eIF4G^{HEAT1} homolog (the middle domain of eIF4GII, PDB ID: 1HU3) and typical RNA binding proteins. The RNA recognition motif (RRM) from human antigen R (PDB ID: 6GC5), the arginine rich motif from HIV Rev protein (PDB ID: 4PMI), and the double stranded RNA binding domain (dsRBD) from Staufen1 (PDB ID: 6HTU) are shown as surface where the bound RNAs are shown as violet sticks. Glu and Asp residues are colored red, Lys and Arg residues are colored blue, and Phe and Tyr residues are colored green. For RRM, arginine binding motif, and dsRBD, RNA binding surfaces are marked by dashed orange lines. The acidic residues, Glu and Asp, are rarely found on RNA–protein interfaces, while the basic and aromatic residues, Lys, Arg, Tyr, and Phe, are frequently found on RNA–protein interfaces. There is no surface area on eIF4G^{HEAT1} that is convincing as the RNA-binding site[1], as corroborated by the structure- and sequence-based predictions[2].

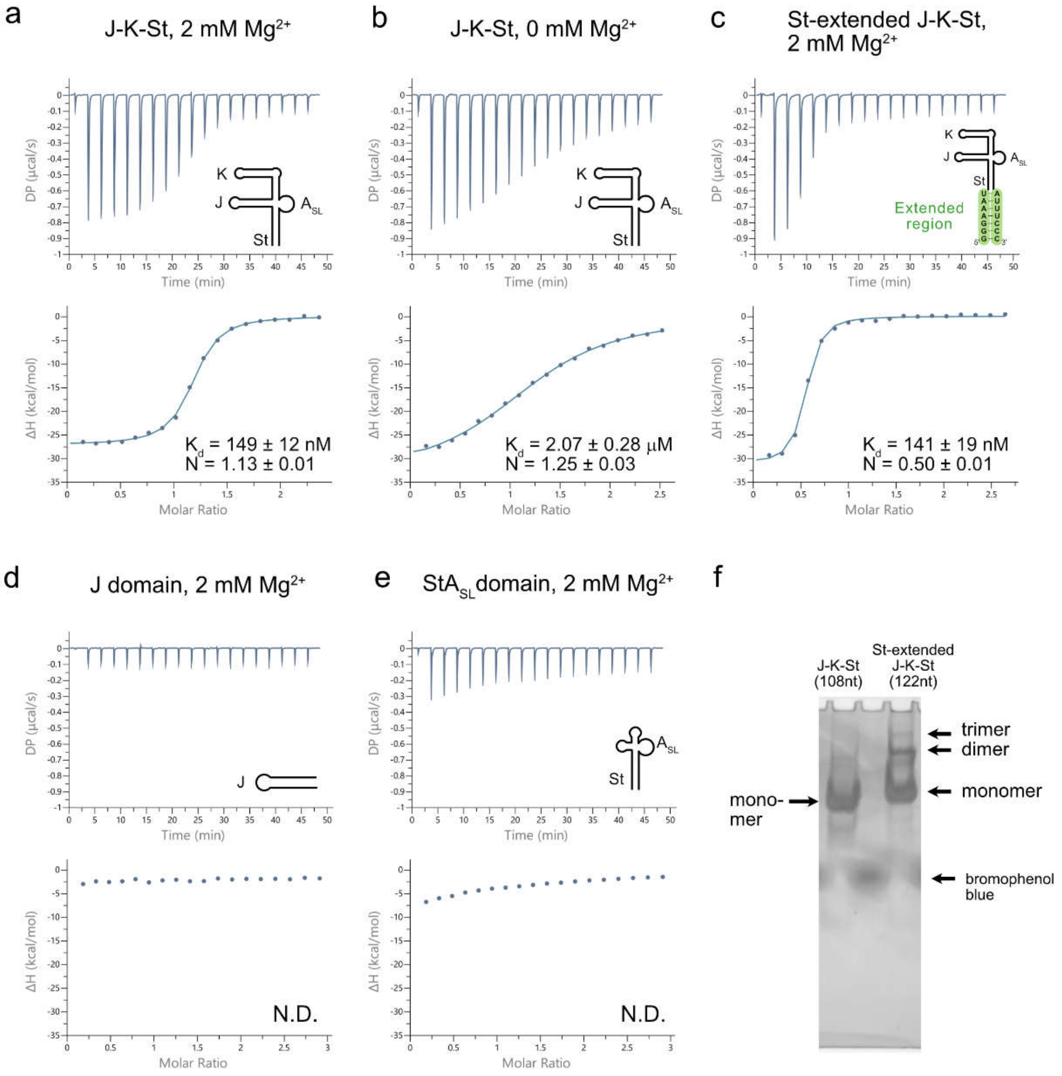

Supplementary Fig. 2 | ITC experiments

(a, b) ITC data of the interaction between J-K-St and eIF4G^{HEAT1}, in the presence of 2 mM Mg^{2+} (a) and in the absence of Mg^{2+} (b).

(c) ITC data of the interaction between St-extended J-K-St and eIF4G^{HEAT1}, in the presence of 2 mM Mg^{2+}. Extended regions at the terminus of the St domain are schematically shown in green in the inset. See also (f) for the reduced stoichiometry.

(d) ITC data of the interaction between the J domain and eIF4G^{HEAT1}, in the presence of 2 mM Mg^{2+}. N.D., not determined. See Supplementary Fig. 6a for the design of the J domain.

(e) ITC data of the interaction between the StA$_{SL}$ domain and eIF4G^{HEAT1} in the presence of 2 mM Mg^{2+}. N.D., not determined. See Supplementary Fig. 6a for the design of the StA$_{SL}$ domain.

(f) Native polyacrylamide gel electrophoresis (PAGE) analysis of J-K-St and St-extended J-K-St. Bands corresponding to the dimer and trimer were observed only for St-extended J-K-St. These

multimers, formed due to misfolding, decrease the effective concentration of the variant with respect to that calculated from the absorbance at 260 nm, resulting in the reduced stoichiometry (N = 0.50 ± 0.01) observed in (c). It should be noted here that discrepancy of the effective concentration of the sample in the ITC cell (i.e., the RNA) does not affect the K_d values obtained in the one-site fitting model employed here. The misfolded molecules were not included in the cryo-EM analyses, as the samples were purified before the cryo-EM analyses, ensuring that only the RNA molecules that formed a complex with $eIF4G^{HEAT1}$/eIF4A were used.

N.D., not determined. The ITC experiment (a) was repeated three times with similar results. The ITC experiments (b-e) were conducted at least two times with similar results. The ±values indicate the standard error of the fitting.

Source data are provided as a Source Data file.

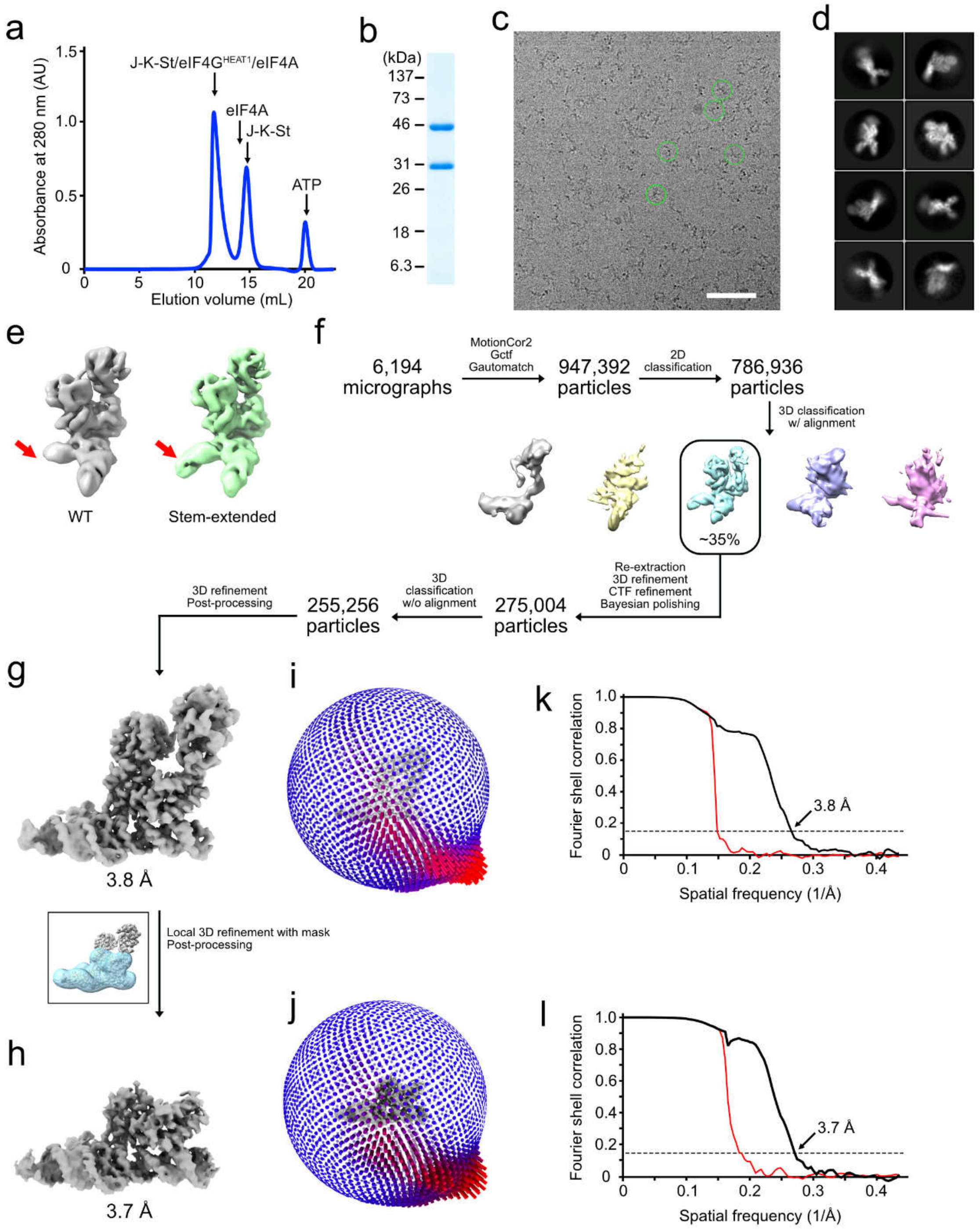

Supplementary Fig. 3 | Cryo-electron microscopy and image processing

(a) Size exclusion chromatography profile of the J-K-St/eIF4G^{HEAT1}/eIF4A ternary complex. The positions corresponding to the elutions of the complex and free components are labeled and indicated by arrows.

(b) SDS-PAGE analysis of the peak fraction corresponding to the ternary complex. Proteins in the gel were stained with Coomassie Brilliant Blue G-250. Source data are provided as a Source Data file.
(c) Representative cryo-EM micrograph of the vitrified J-K-St/eIF4G^{HEAT1}/eIF4A complex. Green circles indicate examples of individual particles. Scale bar is 50 nm.
(d) Selected 2D-class averages of the ternary complex.
(e) Low-pass-filtered Cryo-EM maps obtained from the pilot data of the ternary complexes containing WT J-K-St (left, grey) or St-extended J-K-St (right, green), collected on JEM-Z300CF. Red arrows indicate the region of an extra density observed only in the St-extended J-K-St/eIF4G^{HEAT1}/eIF4A ternary complex.
(f) Cryo-EM data-processing workflow for the St-extended J-K-St/eIF4G^{HEAT1}/eIF4A ternary complex collected on JEM-Z320FHC.
(g and h) The refined cryo-EM map of the whole complex (g) and the refined map with local alignment and a mask (inset) focused on the region of J-K-St/eIF4G^{HEAT1} (h).
(i and j) Angular distribution plot of all particles that contributed to the refined map of the whole complex (i) and the J-K-St/eIF4G^{HEAT1}-focused map (j).
(k and l) Gold-standard Fourier shell correlation (FSC) curves for the whole complex map (k) and the J-K-St/eIF4G^{HEAT1}-focused map (l) after correction for masking effects (black line). The phase randomized FSC curves are colored red. The map resolutions were estimated based on the FSC = 0.143 criterion.

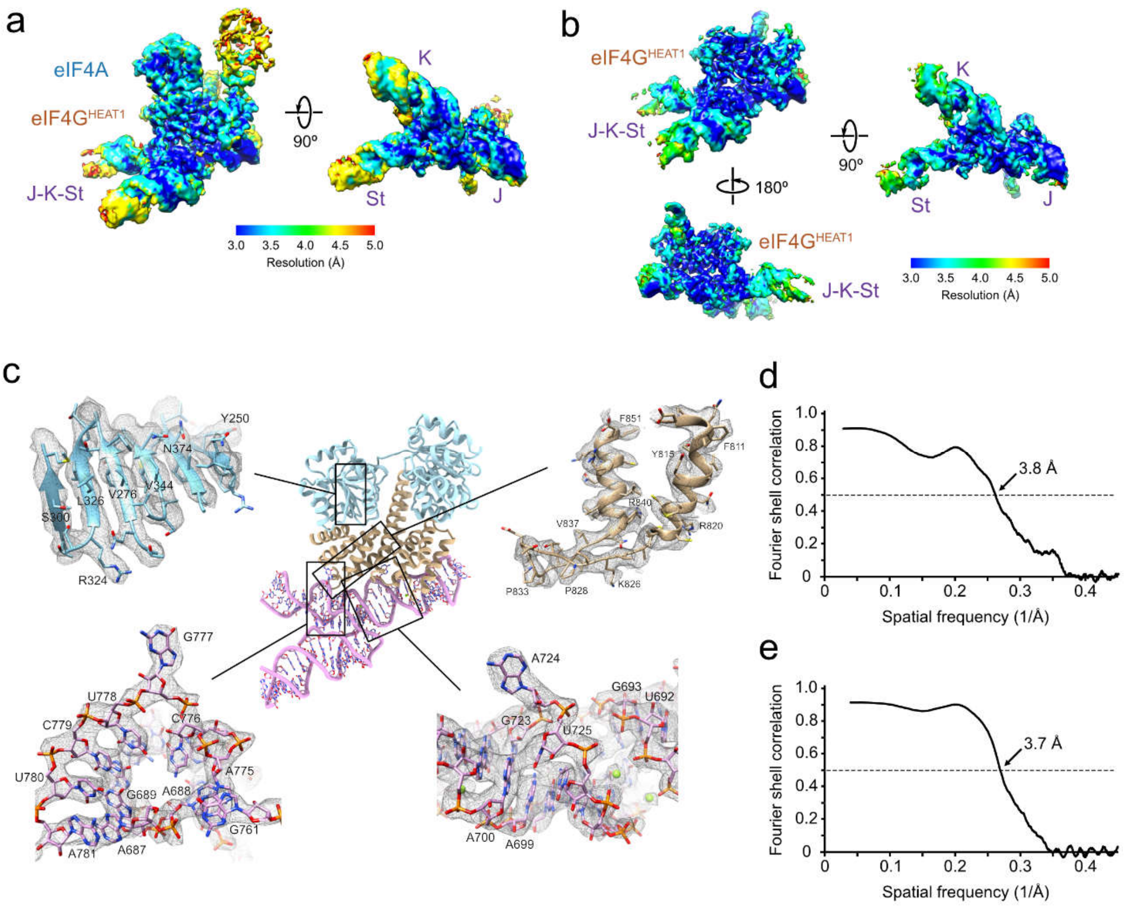

Supplementary Fig. 4 | Cryo-electron microscopy map analyses

(a and b) Local resolutions for the whole complex map (a) and the focused map (b) were calculated using ResMap.

(c) Cryo-EM densities and refined models for selected regions at the St/A_{SL} domain (lower left) and the J domain (lower right) of J-K-St RNA, eIF4G^{HEAT1} (upper right), and eIF4A (upper left) in the ternary complex.

(d and e) Cross-validation FSC curves of the refined models versus the final maps. The correlation is above 0.5 up to resolutions of 3.8 Å and 3.7 Å for the ternary complex (d) and the focused region of J-K-St/eIF4G^{HEAT1} (e), respectively.

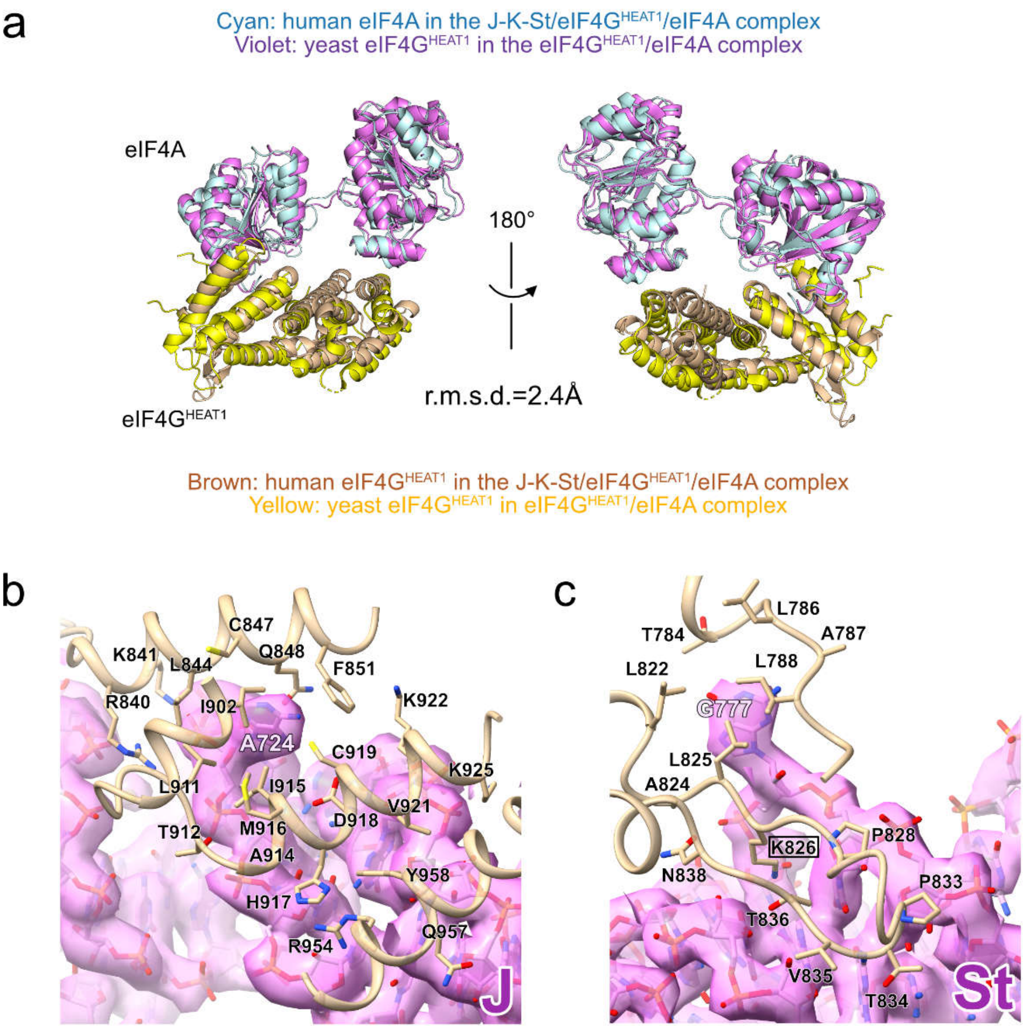

Supplementary Fig. 5 | Analyses of the J-K-St/eIF4G^{HEAT1}/eIF4A structure

(a) Structural alignment of the eIF4G^{HEAT1}/eIF4A complex in the absence and presence of J-K-St. The human eIF4G^{HEAT1} (brown) / human eIF4A (cyan) dimer from the J-K-St/ eIF4G^{HEAT1}/eIF4A ternary complex (this study) is overlaid with the yeast eIF4G^{HEAT1} (yellow) / yeast eIF4A (violet) dimer (PDB ID: 2VSO). Even though the primary sequences are different, with sequence identities of 64.2% for eIF4A and 28.8% for eIF4G^{HEAT1}, the r.m.s.d. value of the two dimers is as low as 2.4 Å, indicating that the structural rearrangement upon the interaction with J-K-St is small.

(b) eIF4G^{HEAT1} residues interacting with the J domain in the J-K-St/eIF4G^{HEAT1}/eIF4A complex. Cryo-EM map of the J domain from J-K-St is shown as a purple surface, where the nucleobase extruding from the J domain, A724, is labeled, and the model the J-K-S RNA is shown in stick representation. A part of the eIF4G^{HEAT1} backbone structure is shown in cartoon representation (brown), and the side chains at the RNA-protein interface are shown in stick representation.

(c) eIF4G^{HEAT1} residues interacting with the St domain in the J-K-St/eIF4G^{HEAT1}/eIF4A complex. Cryo-EM map of the St domain from J-K-St is shown as a purple surface, where the nucleobase extruding from the St domain, G777, is labeled, and the model the J-K-S RNA is shown in stick representation. A part of the eIF4G^{HEAT1} backbone structure is shown in cartoon representation (brown), and the side chains at the RNA-protein interface are shown in stick representation. Lys826, whose side chain is inserted within the cavity formed by the flipping-out of G777, is highlighted by a box.

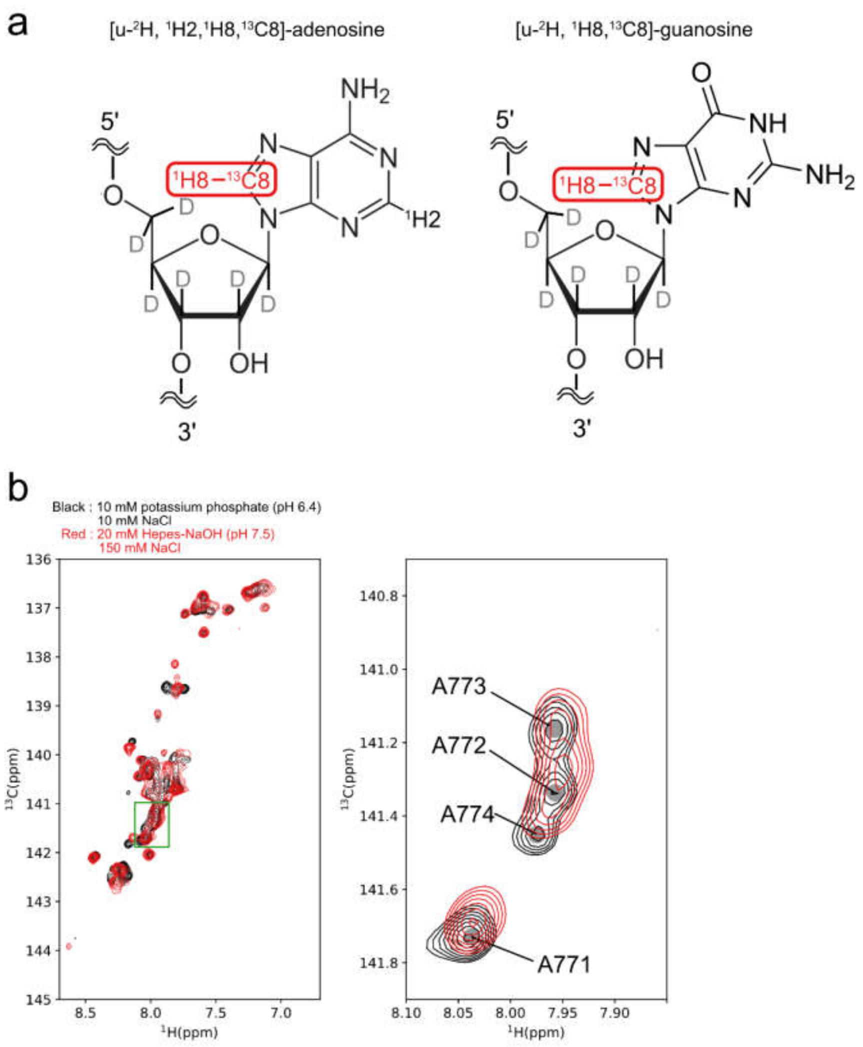

Supplementary Fig. 6 | Site-selective isotope labeling and buffer effects in NMR analyses

(a) Adenosine (left) and guanosine (right) are site-specifically isotope-labeled at the C8 positions, whereas the riboses are perdeuterated, thus alleviating the strong inter- and/or intra-residual dipole-dipole interactions that broaden the NMR signals. The ribose and nucleobase moieties of uridines and cytosines used in this study are perdeuterated.

(b) ^{1}H8-^{13}C8 aromatic TROSY spectra of [u-^{2}H, {^{1}H2,^{1}H8,^{13}C8}-Ade, {^{1}H8,^{13}C8}-Gua] labeled J-K-St. The spectra acquired in buffer containing 10 mM potassium chloride (pH 6.4) and 10 mM NaCl, and in buffer containing 20 mM Hepes-NaOH (pH 7.5), 150 mM NaCl are shown in black and red, respectively. Although several signals exhibited chemical shift perturbation or broadening of the line widths between these two buffer conditions, more than 80% of the observed signals overlapped, indicating that overall secondary structures are virtually identical. On the right, the expanded region containing signals from the A_{SL} domain, marked as green box, are shown. A_{SL} domain signals are not largely perturbed between these two pH and salt concentration conditions.

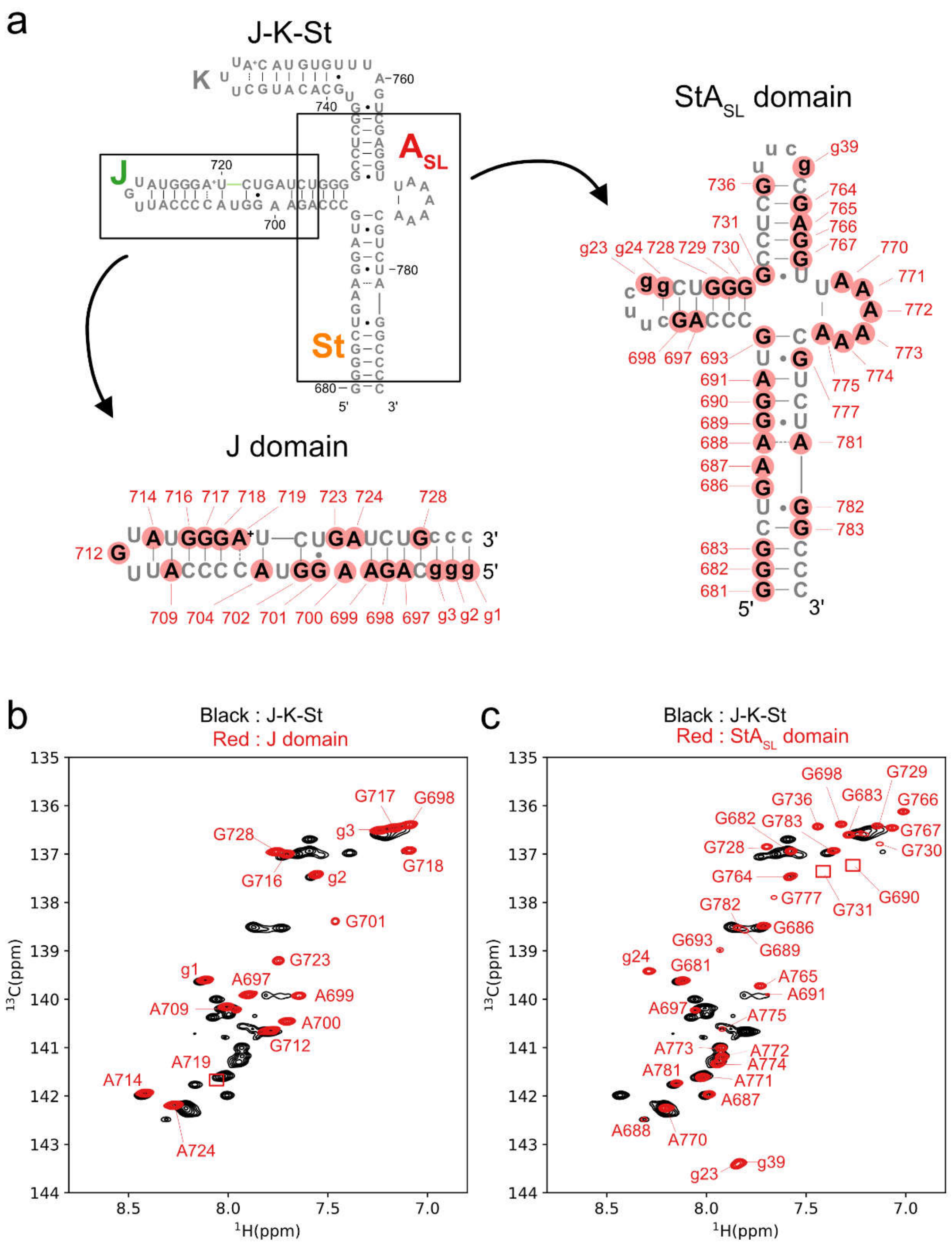

Supplementary Fig. 7 | Construction of the J and StA$_{SL}$ domains

(a) Design of the J and StA$_{SL}$ domains. The nucleotides that are not present in the full-length J-K-St are shown in lower cases. Adenosine and guanosine bases that are observed in the NMR experiments are highlighted with red circles. For the J domain, the 5′ terminal bases are substituted with guanosine bases to facilitate transcription by T7 RNA polymerase. This construct is identical to the SL-J construct

used in the previous study[3]. For the StA_{SL} domain, the tips of the J and K domains are substituted with UUCG tetraloops. This construct is similar to the ΔJΔK construct used in the previous study, in which GAGA tetraloops were used instead of UUCG tetraloops.

(b and c) Overlay of the ^{1}H-^{13}C aromatic TROSY spectra of the J domain (b) and the StA_{SL} domain (c), shown in red, with that of the full-length J-K-St shown in black. Assignments for the isolated domains are shown. For the weak signals that are below the threshold, red boxes are shown to label their positions. Overlapping of the signals from the isolated domains, J and StA_{SL}, with those from J-K-St indicates the structural integrities of the domains, as shown previously[3].

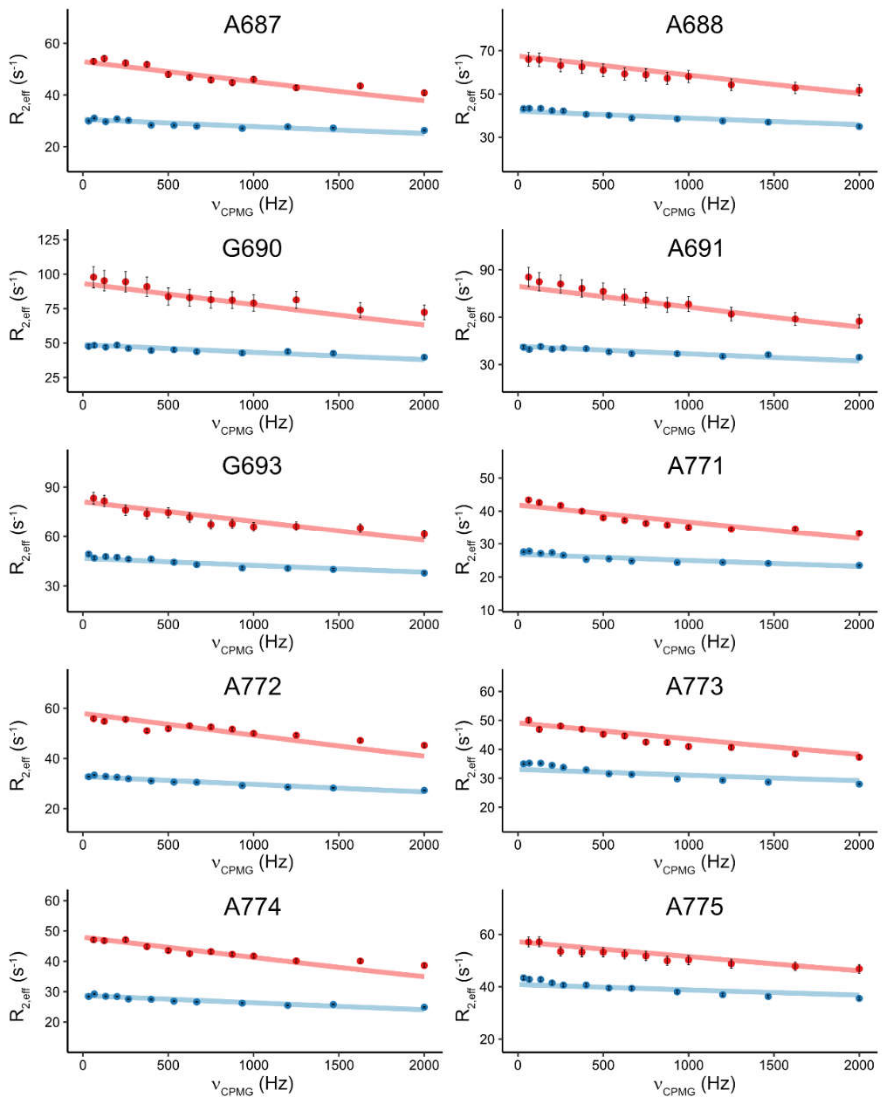

Supplementary Fig. 8 | Global fitting analyses of the relaxation dispersion experiment of the StA_{SL} domain

^{13}C SQ relaxation dispersion data obtained at the ^{13}C frequencies of 150 and 250 MHz are plotted as blue and red points, respectively. These bases simultaneously fit with a 2-site fast exchange model[4], using the identical exchange rates of the two conformations in the equilibrium. The simultaneous fitting indicates that the conformational exchanges of these bases occur in a cooperative manner. Error bars indicate experimental errors derived from the signal-to-noise ratio of each correlation, as written in Methods. Source data are provided as a Source Data file.

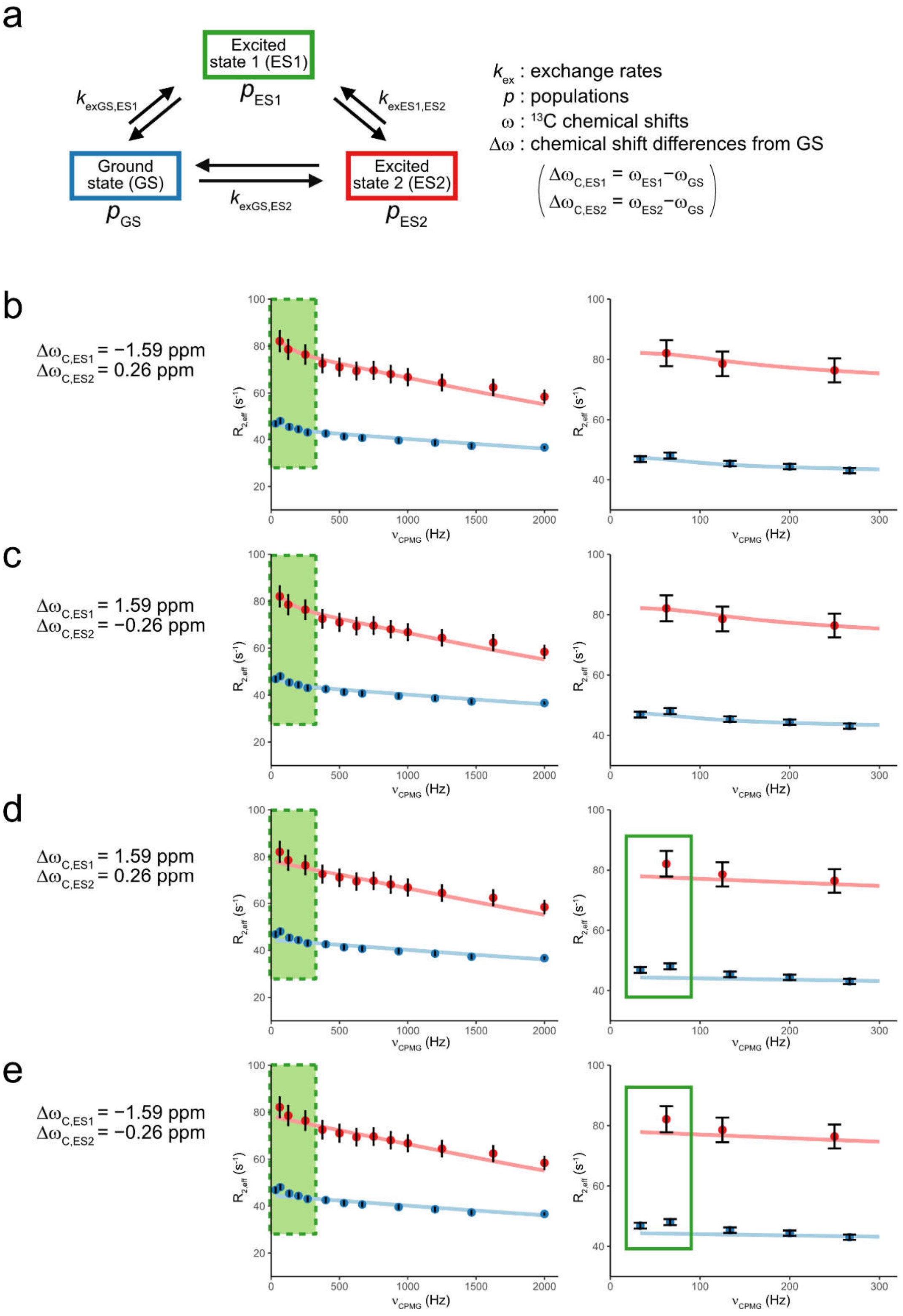
a
Excited state 1 (ES1)
p_ES1
Ground state (GS)
p_GS
Excited state 2 (ES2)
p_ES2
k_exGS,ES1
k_exES1,ES2
k_exGS,ES2
k_ex : exchange rates
p : populations
ω : 13C chemical shifts
Δω : chemical shift differences from GS
Δω_C,ES1 = ω_ES1−ω_GS
Δω_C,ES2 = ω_ES2−ω_GS
b
Δω_C,ES1 = −1.59 ppm
Δω_C,ES2 = 0.26 ppm
c
Δω_C,ES1 = 1.59 ppm
Δω_C,ES2 = −0.26 ppm
d
Δω_C,ES1 = 1.59 ppm
Δω_C,ES2 = 0.26 ppm
e
Δω_C,ES1 = −1.59 ppm
Δω_C,ES2 = −0.26 ppm
R_2,eff (s^-1)
ν_CPMG (Hz)

Supplementary Fig. 9 | Effect of the signs in the chemical shift differences on the relaxation dispersion curves from G777

(a) Definitions of parameters used in the three-state exchange.

(b-e) The calculated dispersion curves for the three-state exchange of G777 are shown as lines, with experimentally obtained $R_{2,eff}$ values shown as points. Data shown in red are at the ^{13}C frequency of 250 MHz, whereas data shown in blue are at 150 MHz. Error bars indicate experimental errors derived from the signal-to-noise ratio of each correlation, as written in Methods. (b) $\Delta\omega_{C,ES1} = -1.59$ ppm, $\Delta\omega_{C,ES2} = 0.26$ ppm, (c) $\Delta\omega_{C,ES1} = 1.59$ ppm, $\Delta\omega_{C,ES2} = -0.26$ ppm, (d) $\Delta\omega_{C,ES1} = 1.59$ ppm, $\Delta\omega_{C,ES2} = 0.26$ ppm, (e) $\Delta\omega_{C,ES1} = -1.59$ ppm, $\Delta\omega_{C,ES2} = -0.26$ ppm. The other parameters, $p_{ES1} = 0.183$, $p_{ES2} = 0.017$, $k_{exGS,ES1} = 18{,}100\ s^{-1}$, $k_{exGS,ES2} = 163\ s^{-1}$, and $k_{exES1,ES2} = 630\ s^{-1}$, are identical for all the four panels. On the right, the expanded region of ν_{CPMG} of 0-300 Hz (shown as green dashed boxes on the left) are shown. Reversing the signs of $\Delta\omega_{C,ES1}$ and $\Delta\omega_{C,ES2}$ at the same time retains the curve indistinguishable (b and c), whereas reversing the signs of either of $\Delta\omega_{C,ES2}$ (b and d) or $\Delta\omega_{C,ES1}$ (b and e) changes the curves, and the calculated curves differ from the experimental data points (d and e, green open boxes on the right). These results demonstrate that $\Delta\omega_{C,ES1}$ and $\Delta\omega_{C,ES2}$ should have different signs, but it is not possible to distinguish which one has the negative sign. Source data are provided as a Source Data file.

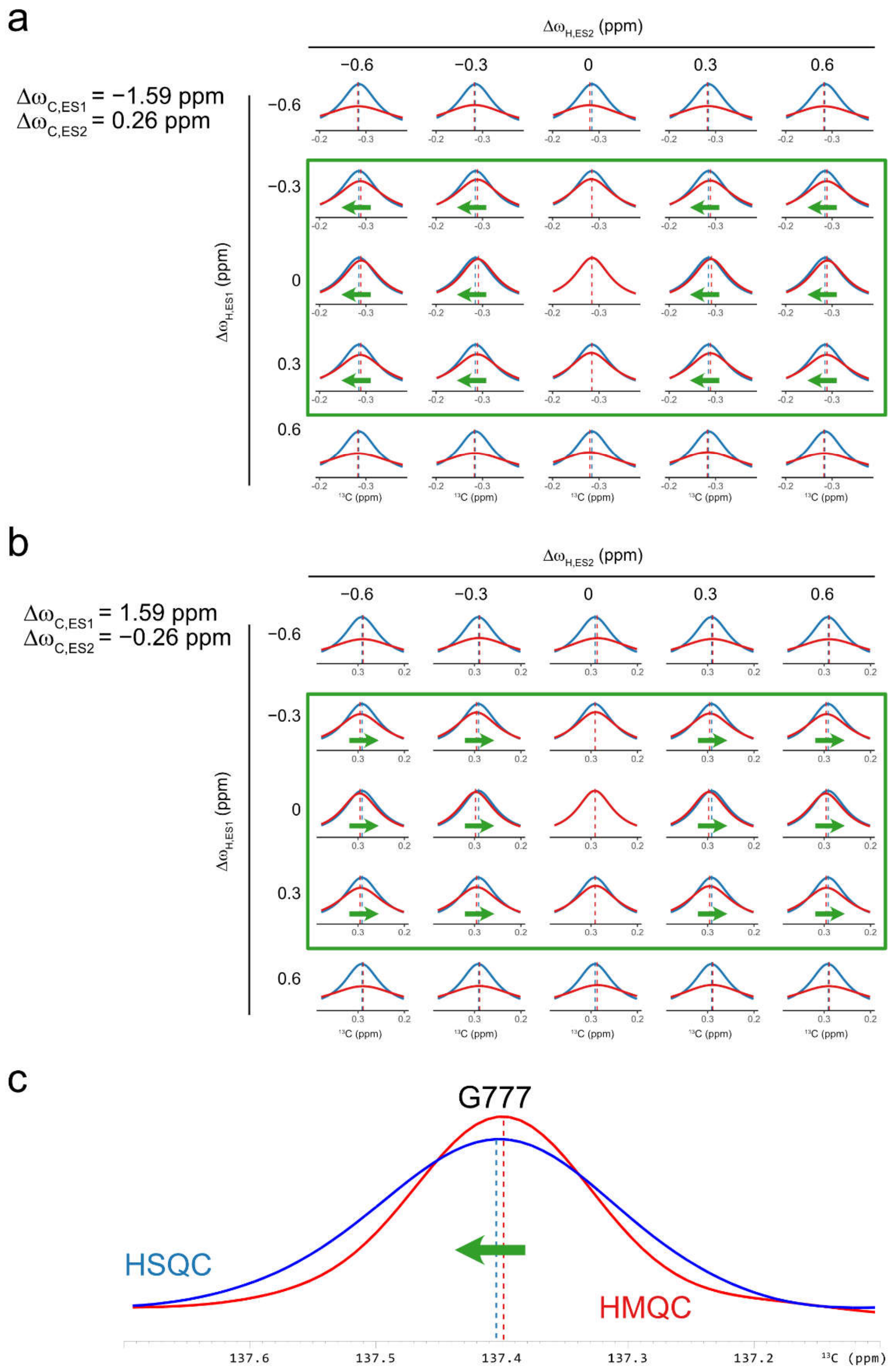

Supplementary Fig. 10 | Determination of the signs of the chemical shift differences by comparing HSQC and HMQC signals

(a and b) Simulated ^{13}C line shapes of the signals in the indirect dimension of the heteronuclear single-quantum coherence (HSQC, blue) and heteronuclear multiple quantum coherence (HMQC, red) spectra at the ^{13}C frequency of 150 MHz. Calculations were conducted by using the parameters p_{ES1} = 0.183, p_{ES2} = 0.017, $k_{exGS,ES1}$ = 18,100 s^{-1}, $k_{exGS,ES2}$ = 163 s^{-1}, and $k_{exES1,ES2}$ = 630 s^{-1}, where $\Delta\omega_{C,ES1}$ = −1.59 ppm and $\Delta\omega_{C,ES2}$ = 0.26 ppm (a), or $\Delta\omega_{C,ES1}$ = 1.59 ppm, $\Delta\omega_{C,ES2}$ = −0.26 ppm (b). For calculation of the line shape in the HMQC spectra, chemical shift differences of the ES1 and ES2 from GS in the ^{1}H dimension, $\Delta\omega_{H,ES1}$ and $\Delta\omega_{H,ES2}$, are systematically varied to be −0.6, −0.3, 0, 0.3, or 0.6 ppm. Green boxes indicate the conditions where HMQC signals are not broadened more than twice than HSQC signals. Within the conditions within the green boxes, HSQC signals would be observed at the lower field when $\Delta\omega_{C,ES1}$ = −1.59 ppm and $\Delta\omega_{C,ES2}$ = 0.26 ppm (a), whereas at the upper field when $\Delta\omega_{C,ES1}$ = 1.59 ppm, $\Delta\omega_{C,ES2}$ = −0.26 ppm (b). The relative direction of HSQC signals with respect to the HMQC signals are indicated by green arrows.

(c) Experimentally obtained signal of G777 at 30°C and at the ^{13}C frequency of 150 MHz. The HSQC signal was observed 0.01 ppm lower field with respect to the HMQC signal, indicating that $\Delta\omega_{C,ES1}$ = −1.59 ppm and $\Delta\omega_{C,ES2}$ = 0.26 ppm (a) is correct.

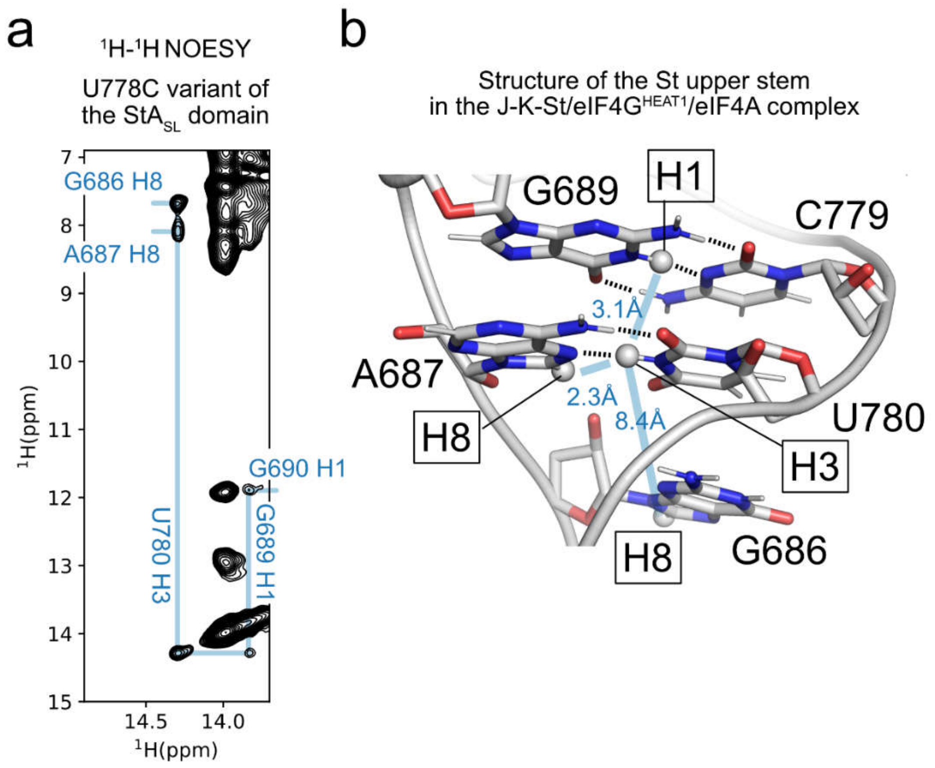

Supplementary Fig. 11 | Secondary structure analyses of the U778C variant of the StA_{SL} domain

(a) A region from the ^{1}H-^{1}H NOESY spectrum of the U778C variant of StA_{SL} at 10°C, showing the NOE signals observed for the St upper stem region.

(b) A magnified view of the structure of the upper stem of the St domain in the J-K-St/eIF4G^{HEAT1}/eIF4A complex. U780 H3 is close to A687 H8, G689 H1, and G686 H8. This structure corresponds to the NOE pattern observed for the U778C variant in the absence of eIF4G^{HEAT1}, at 10°C, except for the relatively longer distance between U780 H3 and G686 H8, indicating that U780 is base-paired with A687 for the U778C variant of StA_{SL}, at 10°C. The NOE signal between H3 of U778 and G686 H8 suggests that the relative orientation between these two bases is different from that in the structure of the complex, but supports that U778 is base-paired with A687.

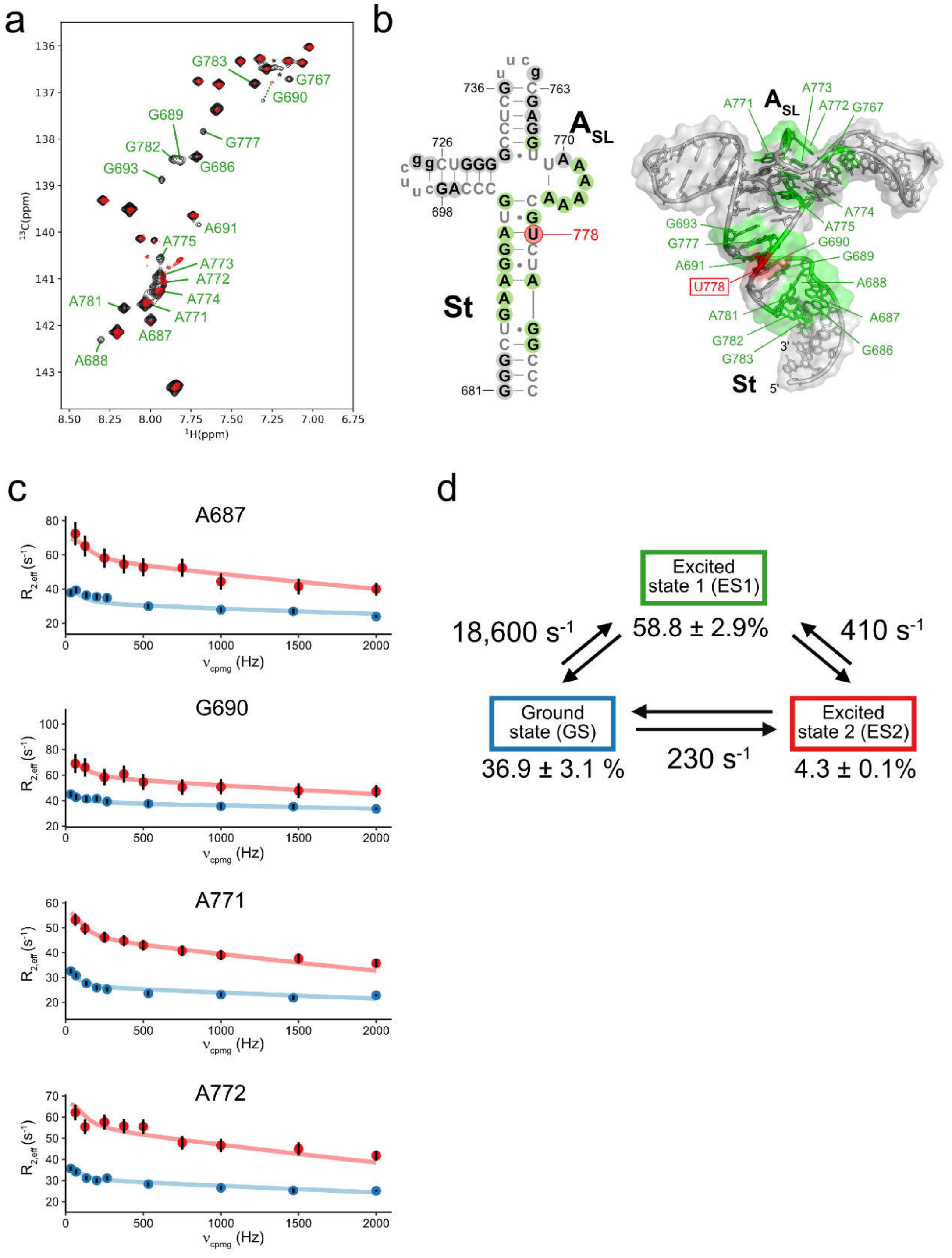

Supplementary Fig. 12 | Relaxation dispersion analyses of the U778C variant of the StA_{SL} domain (a) Overlay of the $^{1}H8$-$^{13}C8$ aromatic TROSY spectra of the wild-type StA_{SL} domain (black) and its U778C variant (red). Signals broadened or exhibited chemical shift perturbation are labeled. The other signals are not largely perturbed, indicating that the structure is not deteriorated by the introduction of

the U778C mutation. Asterisks indicate unassigned minor peaks.

(b) Mapping of the perturbed signals onto the secondary structure (left) or tertiary structure model (right) of the StA_{SL} domain. Red: U778, green: broadened or chemical shift-perturbed bases. These perturbed bases are located widely in the St and A_{SL} domains, indicating that the U778C mutation perturbed the cooperative conformational equilibrium within these domains.

(c) Relaxation dispersion curve fitting. $R_{2,eff}$ values obtained at 30°C and at the ^{13}C frequencies of 250 MHz and 150 MHz are shown by red and blue points, respectively. The relaxation dispersion profiles were globally fit with the 3-site exchange model with the exchange rates obtained for G777 in the wild-type (Fig. 4c), while assuming that the chemical shift differences between ES1 and ES2 are smaller than 0.2 ppm. A representative fitted curves are shown as red and blue lines for the ^{13}C frequencies of 250 MHz and 150 MHz, respectively. Error bars indicate experimental errors derived from the signal-to-noise ratio of each correlation, as written in Methods. Source data are provided as a Source Data file.

(d) Exchange parameters obtained from the fitting. Parameters obtained from the global fitting of the relaxation dispersion curves of A687, G690, A771, and A772 in the U778C variant (c), with the exchange rates fixed as those from the analyses in the wild-type (Fig. 4c).

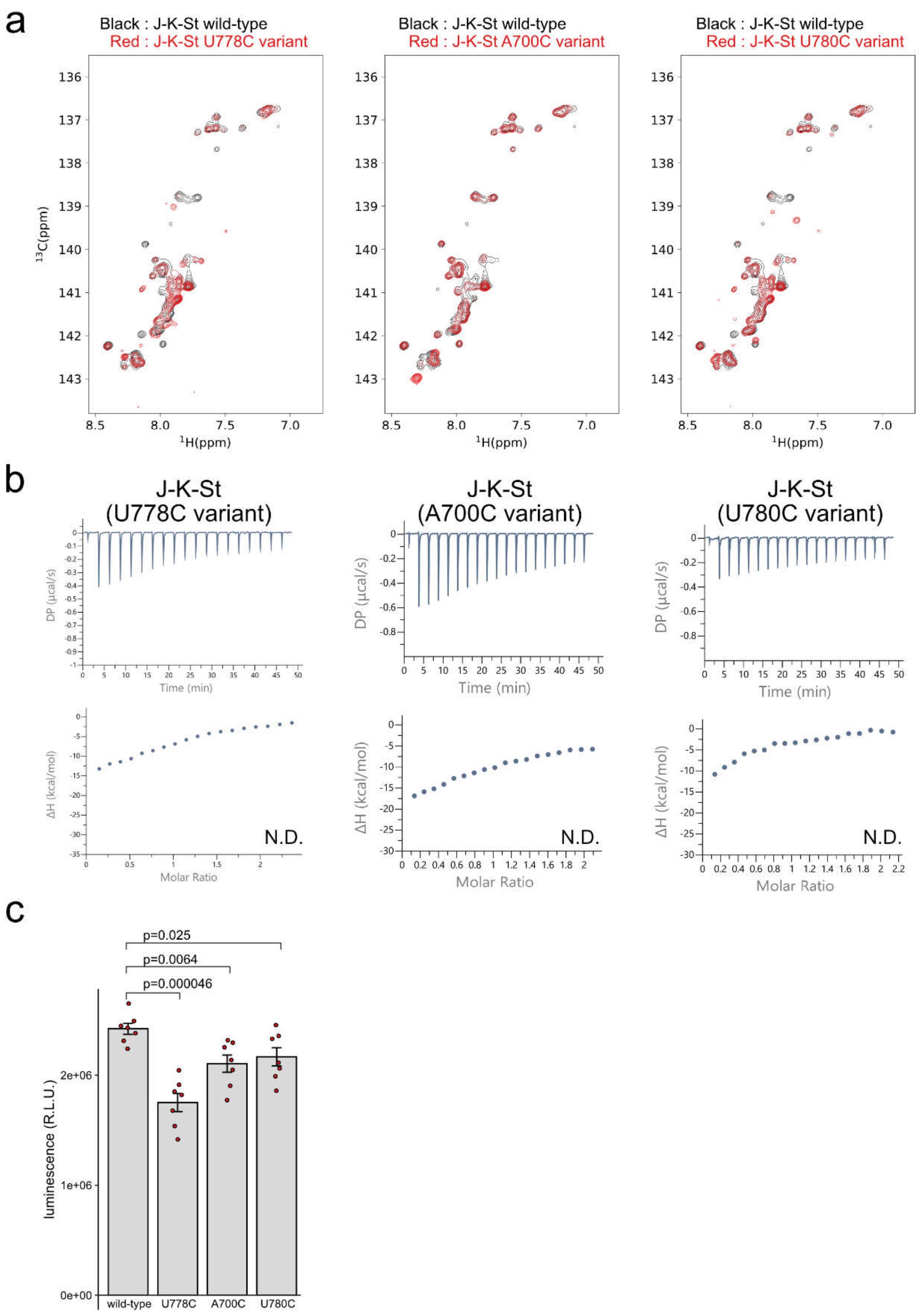

a
Black : J-K-St wild-type
Red : J-K-St U778C variant
Black : J-K-St wild-type
Red : J-K-St A700C variant
Black : J-K-St wild-type
Red : J-K-St U780C variant
13C(ppm)
1H(ppm)
b
J-K-St (U778C variant)
J-K-St (A700C variant)
J-K-St (U780C variant)
DP (µcal/s)
Time (min)
ΔH (kcal/mol)
Molar Ratio
N.D.
c
p=0.025
p=0.0064
p=0.000046
luminescence (R.L.U.)
wild-type
U778C
A700C
U780C

Supplementary Fig. 13 | Correlation between the NMR-observed dynamics and function

(a) Overlay of the ^{1}H8-^{13}C8 aromatic TROSY spectra of the J-K-St U778C (left), A700C (middle), and U780C (right) variants with the corresponding wild-type J-K-St. All spectra were acquired at 35°C and at the ^{1}H frequency of 900 MHz. Although the signals observed for J-K-St are not assigned, more than 80% of the signals observed for the variants are overlaid with those from the wild-type, indicating that the overall secondary structures of these variants are not disrupted by the mutation.

(b) ITC experiments of the J-K-St variants. U778C, A700C, and U780C variants exhibited a lower affinity (K_d value larger than 10 μM) for eIF4G^{HEAT1}, compared to the wild-type (K_d = 149 ± 12 nM). N.D., not determined. The ITC experiments were conducted at least two times with similar results. Source data are provided as a Source Data file.

(c) *In vitro* translation assay. β-galactosidase assay using a human cell-derived in vitro coupled transcription/translation system (n=7 independent replicates). Total amounts of β-galactosidase translated from the EMCV IRES-mediated translation were quantified as luminescence emitted as a result of β-galactosidase reaction. Data are presented as mean ± standard error of the mean (s.e.m.). Statistical significance was determined by two-tailed unpaired Student's t test. Source data are provided as a Source Data file.

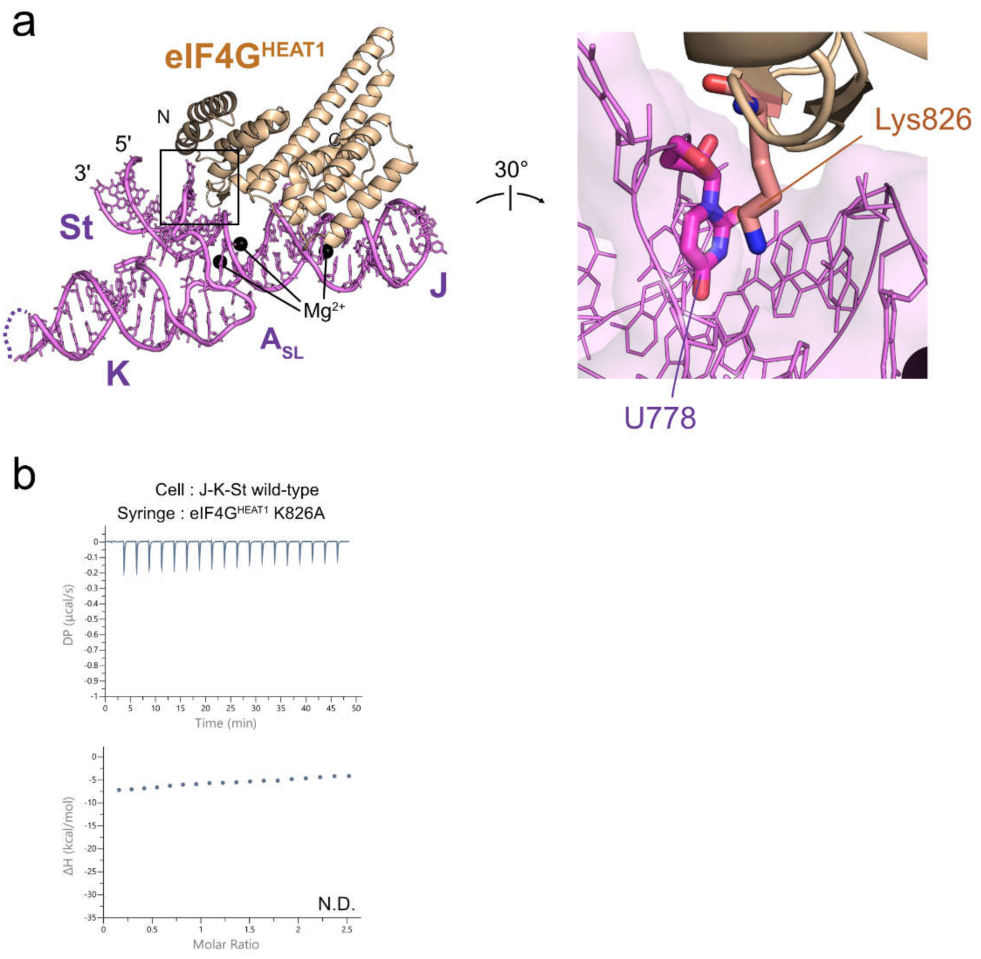

Supplementary Fig. 14 | Interaction of U778 in J-K-St and Lys826 in eIF4G^{HEAT1}

(a) Close-up view of the U778-Lys826 interaction. The side chain of Lys826 of eIF4G^{HEAT1} is extruded to stack onto the pyrimidine ring of U778.

(b) The ITC experiment of the J-K-St wild-type and eIF4G^{HEAT1} K826A. N.D., not determined (K_d larger than 10 μM). The ITC experiment was conducted at least two times with similar results. Source data are provided as a Source Data file.

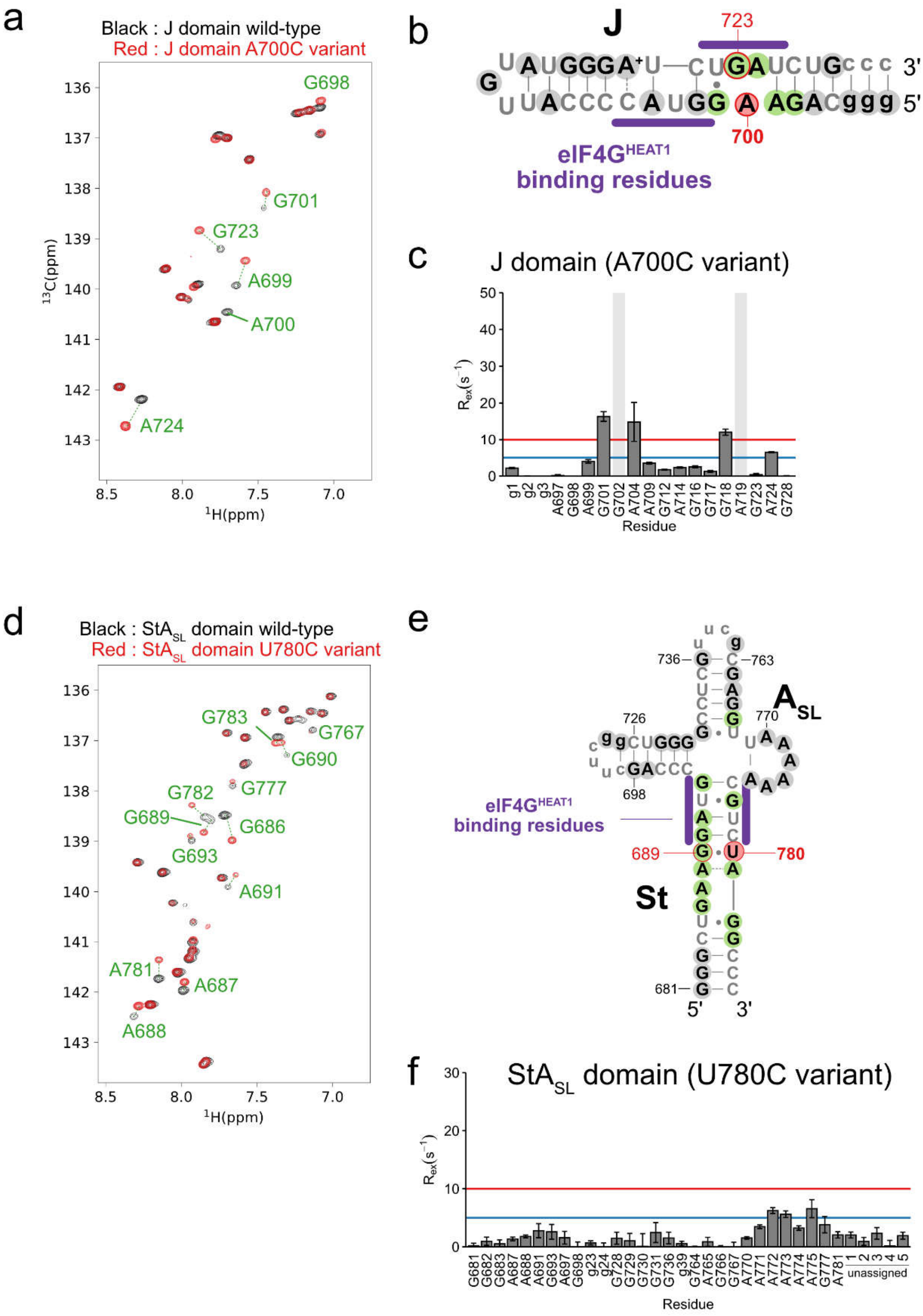

a
Black : J domain wild-type
Red : J domain A700C variant
G698
G701
G723
A699
A700
A724
13C(ppm)
1H(ppm)
b
J
723
700
eIF4GHEAT1
binding residues
c
J domain (A700C variant)
Rex(s-1)
Residue
d
Black : StASL domain wild-type
Red : StASL domain U780C variant
G783
G767
G690
G777
G782
G689
G686
G693
A691
A781
A687
A688
e
ASL
St
eIF4GHEAT1
binding residues
689
780
f
StASL domain (U780C variant)
Rex(s-1)
unassigned
Residue

Supplementary Fig. 15 | Suppression of the conformational dynamics by mutations

(a) Overlay of the ^{1}H8-^{13}C8 aromatic TROSY spectra of the J domain A700C variant (red) with the wild-type (black). All spectra were acquired at 30°C and at the ^{1}H frequency of 800 MHz. The signals perturbed by the mutation are indicated with green labels.

(b) Mapping of the bases perturbed by the A700C mutation on the secondary structure. A700C mutation is designed to form a base pair with G723, suppressing the conformational dynamics observed in the J domain bulge, without substituting the bases that directly interact with eIF4G^{HEAT1} (highlighted with purple lines).

(c) R_{ex} analyses of the A700C variant of the J domain. R_{ex} values observed for the J domain bases are largely suppressed by the mutation (see Fig. 3a for wild type), indicating that the conformational dynamics in the J domain are suppressed. Error bars indicate experimental errors derived from the signal-to-noise ratio of each correlation, as written in Methods. Source data are provided as a Source Data file.

(d) Overlay of the ^{1}H8-^{13}C8 aromatic TROSY spectra of the StA$_{SL}$ domain U780C variant (red) with the wild-type (black). All spectra were acquired at 30°C and at the ^{1}H frequency of 800 MHz. The signals perturbed by the mutation are indicated with green labels.

(e) Mapping of the bases perturbed by the U780C mutation on the secondary structure. U780C mutation is designed to stabilize the base pair with G689, suppressing the register-shift observed in the upper stem of the StA$_{SL}$ domain, without substituting the bases that directly interact with eIF4G^{HEAT1} (highlighted with purple lines).

(f) R_{ex} analyses of the U780C variant of the StA$_{SL}$ domain. R_{ex} values observed for the StA$_{SL}$ domain bases are largely suppressed by the mutation (see Fig. 3b for wild type), indicating that the conformational dynamics in the StA$_{SL}$ domain are suppressed. Error bars indicate experimental errors derived from the signal-to-noise ratio of each correlation, as written in Methods. Source data are provided as a Source Data file.

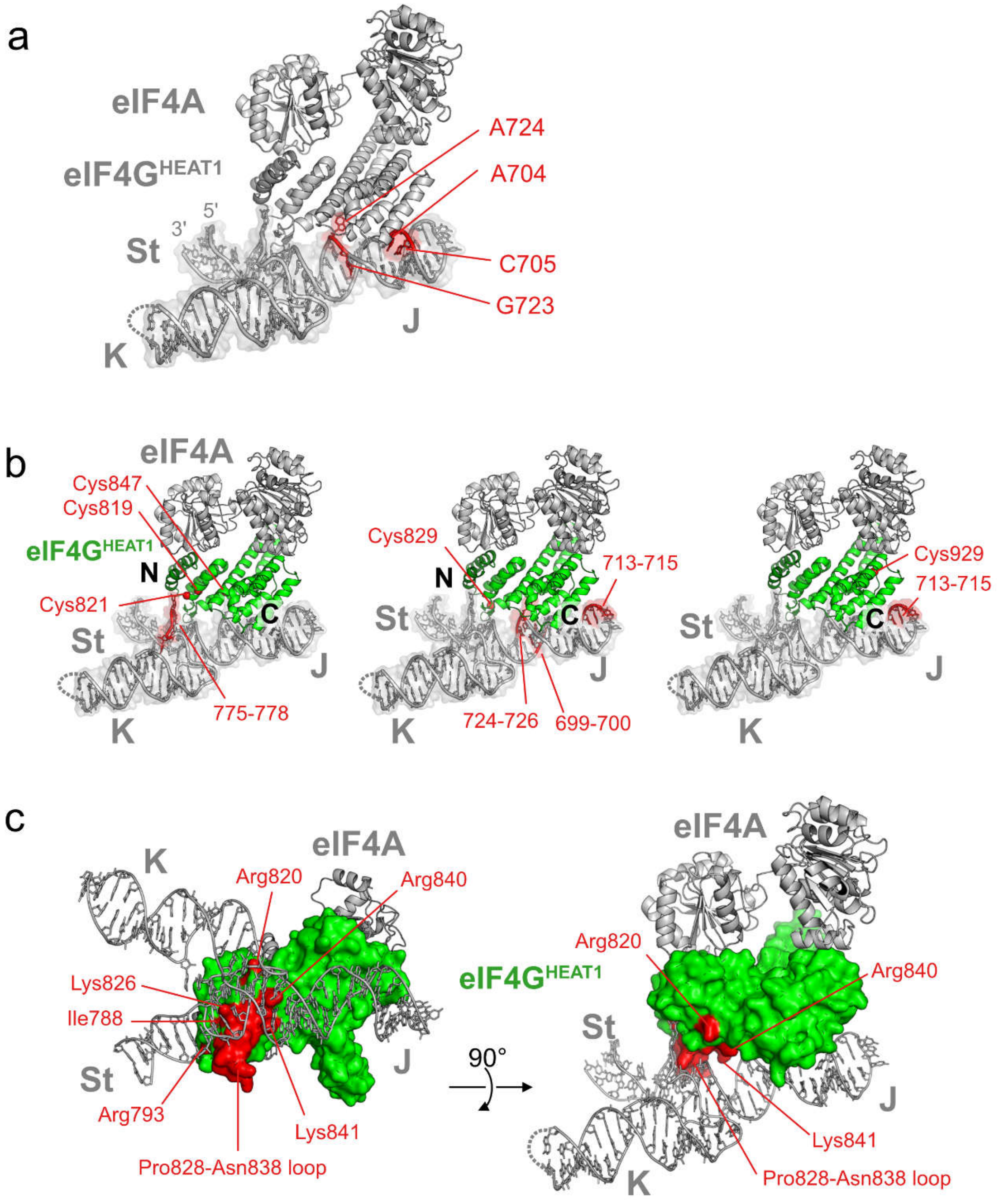

Supplementary Fig. 16 | Comparison with the previous biochemical assays on the interaction between eIF4G^{HEAT1} and J-K-St.

(a) Mapping of the J domain bases identified to be involved in the IRES-dependent translation in the previous literature[5]. The bases in the J domain, A704, C705, G723, and A724, that are shown to be important in the IRES-dependent translation[5] is mapped on the cryo-EM structure of the J-K-St/eIF4G^{HEAT1}/eIF4A complex. These bases are on the interface of the eIF4G^{HEAT1} in the structure, corroborating that these bases play important roles in the interaction with eIF4G^{HEAT1}.

(b) Mapping of the proximity regions identified by directed hydroxyl radial cleavage assay in the previous literature[6]. The residues on the eIF4G HEAT1 domain that were labeled with Fe(II)-BABE tags and the regions cleaved by the hydroxyl radical generated from the tags[6] are mapped on the cryo-EM structure of the J-K-St/eIF4G^{HEAT1}/eIF4A complex. (left) Cys 819, Cys821, and Cys847 on eIF4G^{HEAT1} and bases 775-778 in J-K-St, (middle) Cys 829 on eIF4G^{HEAT1} and bases 699-700, 724-726, and 713-715 in J-K-St, (right) Cys929 on eIF4G^{HEAT1} and bases 713-715 in J-K-St. The structure corresponds to the relative orientation of the eIF4G^{HEAT1} to J-K-St identified by the directed hydroxyl radial cleavage assay, i.e., the N-terminus towards St domain and C-terminus towards J domain.

(c) Mapping of the mutational assay on the eIF4G HEAT1 domain in the previous literature[1]. The residues on eIF4G^{HEAT1} previously identified to be involved in the interaction with J-K-St by the mutational study[1] are mapped onto the cryo-EM structure of the J-K-St/eIF4G^{HEAT1}/eIF4A complex in red. These residues are within, or neighboring to, the interface with the St domain, corroborating that these residues play important roles in the interaction with J-K-St.

Supplementary Table 1 | Cryo-EM data collection and refinement statistics.

	J-K-St/eIF4G^{HEAT1}/eIF4A	J-K-St/eIF4G^{HEAT1} (Focused)
**EMDB ID**	EMD-35041	EMD-36046
**PDB ID**	8HUJ	8J7R
**Data collection**		
Microscope	JEM-Z320FHC	
Detector	K2 Summit	
Pixel size (Å)	0.765	
Defocus range (μm)	-1.0 to -2.5	
Voltage (kV)	300	
Total electron dose (e^- Å^{-2})	68.3	
**Reconstruction**		
Final particle number	255,256	
Pixel size (Å)	1.1475	
Box size (pixels)	200	
Map resolution (Å) (FSC = 0.143)	3.76	3.70
Map sharpening B-factor (Å^{-2})	-110	-132
**Refinement**		
Model composition		
Non-hydrogen atoms	7,143	4,034
Protein residues	621	238
Nucleotide residues	99	97
Water	1	1
Ligands	Mg: 3	Mg: 3
Map CC (mask)	0.78	0.76
Map CC (volume)	0.78	0.76
R.m.s. deviations		
Bond length (Å)	0.005	0.010
Bond angles (º)	1.012	1.340
**Validation**		
MolProbity score	2.2	2.2
All-atom clashscore	13.6	9.2
Ramachandran plot		
Outliers (%)	0.0	0.0
Allowed (%)	3.2	2.5
Favored (%)	96.8	97.5
Rotamer Outliers (%)	2.5	6.85
C-beta deviations (%)	0.0	0.0

Supplementary references

1. Marcotrigiano, J. *et al.* A conserved HEAT domain within eIF4G directs assembly of the translation initiation machinery. *Mol. Cell* **7**, 193–203 (2001).
2. Li, S., Yamashita, K., Amada, K. M. & Standley, D. M. Quantifying sequence and structural features of protein-RNA interactions. *Nucleic Acids Res.* **42**, 10086–98 (2014).
3. Imai, S., Kumar, P., Hellen, C. U. T., D'Souza, V. M. & Wagner, G. An accurately preorganized IRES RNA structure enables eIF4G capture for initiation of viral translation. *Nat. Struct. Mol. Biol.* **23**, 859–64 (2016).
4. Luz, Z. & Meiboom, S. Nuclear Magnetic Resonance Study of the Protolysis of Trimethylammonium Ion in Aqueous Solution—Order of the Reaction with Respect to Solvent. *J. Chem. Phys.* **39**, 366–370 (1963).
5. Clark, A. T., Robertson, M. E. M., Conn, G. L. & Belsham, G. J. Conserved nucleotides within the J domain of the encephalomyocarditis virus internal ribosome entry site are required for activity and for interaction with eIF4G. *J. Virol.* **77**, 12441–9 (2003).
6. Kolupaeva, V. G., Lomakin, I. B., Pestova, T. V & Hellen, C. U. T. Eukaryotic initiation factors 4G and 4A mediate conformational changes downstream of the initiation codon of the encephalomyocarditis virus internal ribosomal entry site. *Mol. Cell. Biol.* **23**, 687–98 (2003).
