# Supplementary material for: Dynamically regulated two-site interaction of viral RNA to capture host translation initiation factor
Source: Nat Commun. 2023 Aug 28;14:4977. doi: 10.1038/s41467-023-40582-6 (PMC10462655; doi:10.1038/s41467-023-40582-6)
Supplement: Supplementary file 3 — Description of Additional Supplementary Files [file 41467_2023_40582_MOESM3_ESM.pdf]

<u>Description of additional supplementary files</u>

Supplementary Data 1: Sequences of the primers used for cloning
