# Supplementary material for: Dynamically regulated two-site interaction of viral RNA to capture host translation initiation factor
Source: Nat Commun. 2023 Aug 28;14:4977. doi: 10.1038/s41467-023-40582-6 (PMC10462655; doi:10.1038/s41467-023-40582-6)
Supplement: Supplementary file 5 — Reporting Summary [file 41467_2023_40582_MOESM5_ESM.pdf]

nature portfolio

Corresponding author(s): Shunsuke Imai, Ichio Shimada

Last updated by author(s): Jul 21, 2023

Reporting Summary

Nature Portfolio wishes to improve the reproducibility of the work that we publish. This form provides structure for consistency and transparency in reporting. For further information on Nature Portfolio policies, see our Editorial Policies and the Editorial Policy Checklist.

Statistics

For all statistical analyses, confirm that the following items are present in the figure legend, table legend, main text, or Methods section.

n/a	Confirmed	
☐	☒	The exact sample size (*n*) for each experimental group/condition, given as a discrete number and unit of measurement
☐	☒	A statement on whether measurements were taken from distinct samples or whether the same sample was measured repeatedly
☐	☒	The statistical test(s) used AND whether they are one- or two-sided<br>*Only common tests should be described solely by name; describe more complex techniques in the Methods section.*
☒	☐	A description of all covariates tested
☒	☐	A description of any assumptions or corrections, such as tests of normality and adjustment for multiple comparisons
☐	☒	A full description of the statistical parameters including central tendency (e.g. means) or other basic estimates (e.g. regression coefficient) AND variation (e.g. standard deviation) or associated estimates of uncertainty (e.g. confidence intervals)
☐	☒	For null hypothesis testing, the test statistic (e.g. *F*, *t*, *r*) with confidence intervals, effect sizes, degrees of freedom and *P* value noted<br>*Give P values as exact values whenever suitable.*
☒	☐	For Bayesian analysis, information on the choice of priors and Markov chain Monte Carlo settings
☒	☐	For hierarchical and complex designs, identification of the appropriate level for tests and full reporting of outcomes
☒	☐	Estimates of effect sizes (e.g. Cohen's *d*, Pearson's *r*), indicating how they were calculated

Our web collection on statistics for biologists contains articles on many of the points above.

Software and code

Policy information about availability of computer code

Data collection	Bruker Topspin 4.1.4 for NMR data collection. Microcal PEAQ-ITC collection 1.41 for ITC data collection. SerialEM version 3.8.9 for cryo-EM data collection.
Data analysis	Bruker Topspin 4.0.8 for NMR data analyses. Microcal PEAQ-ITC analysis 1.41 for ITC data analyses. RELION-3.1, MotionCor2 version 1.4.0, Gctf version 1.18, Gautomatch version 0.56, ResMap version 1.1.4, Coot version 0.9.6, and UCSF Chimera version 1.14, and PHENIX version 1.20.1 for cryo-EM data analyses. The L-BFGS-B algorithm for fitting the relaxation dispersion data fitting is called from the "optim" function in R version 4.2.1. The structures were rendered by using PyMOL version 2.5.0 or UCSF ChimeraX version 1.5.<br>Custom codes used to fit the relaxation dispersion data are available at Zenodo under DOI 10.5281/zenodo.8166504.

For manuscripts utilizing custom algorithms or software that are central to the research but not yet described in published literature, software must be made available to editors and reviewers. We strongly encourage code deposition in a community repository (e.g. GitHub). See the Nature Portfolio guidelines for submitting code & software for further information.

Data

Policy information about availability of data

All manuscripts must include a data availability statement. This statement should provide the following information, where applicable:

- Accession codes, unique identifiers, or web links for publicly available datasets
- A description of any restrictions on data availability
- For clinical datasets or third party data, please ensure that the statement adheres to our policy

The cryo-EM density map and corresponding atomic coordinate of the J-K-St/eIF4GHEAT1/eIF4A complex have been deposited in the Electron Microscopy Data Bank and the Protein Data Bank under accession codes EMD-35041 [https://www.ebi.ac.uk/pdbe/entry/emdb/EMD-35041] and 8HUJ [https://doi.org/10.2210/pdb8HUJ/pdb], respectively. The focused cryo-EM density map and corresponding atomic coordinate of the J-K-St/eIF4GHEAT1 complex have been deposited under accession codes EMD-36046 [https://www.ebi.ac.uk/pdbe/entry/emdb/EMD-36046] and 8J7R [https://doi.org/10.2210/pdb8J7R/pdb], respectively. Other structure data used in this study are available in the Protein Data Bank under accession codes 2NBX [https://doi.org/10.2210/pdb2NBX/pdb], 2NBY [https://doi.org/10.2210/pdb2NBY/pdb], 2NC1 [https://doi.org/10.2210/pdb2NC1/pdb], 6ZMW [https://doi.org/10.2210/pdb6ZMW/pdb], 1HU3 [https://doi.org/10.2210/pdb1HU3/pdb], 6GC5 [https://doi.org/10.2210/pdb6GC5/pdb], 4PMI [https://doi.org/10.2210/pdb4PMI /pdb], 6HTU [https://doi.org/10.2210/pdb6HTU/pdb], and 2VSO [https://doi.org/10.2210/pdb2VSO/pdb]. The chemical shift data of the StASL and J domain have been deposited in Biological Magnetic Resonance Bank under accession codes 51905 [https://doi.org/10.13018/BMR51905] and 51906 [https://doi.org/10.13018/BMR51906], respectively. Chemical shift data used in this study are available from Biological Magnetic Resonance Bank under the accession codes of 25997 [https://doi.org/10.13018/BMR25997] and 26000 [https://doi.org/10.13018/BMR26000]. Protein sequences used in this study are available from Uniprot under accession codes P0A717 [https://www.uniprot.org/uniprotkb/P0A717/entry] (PRPPS), P0A9J6 [https://www.uniprot.org/uniprotkb/P0A9J6/entry] (RK), P69503 [https://www.uniprot.org/uniprotkb/P69503/entry] (APRT), P60546 [https://www.uniprot.org/uniprotkb/P60546/entry] (GMK), P0A9M5 [https://www.uniprot.org/uniprotkb/P0A9M5/entry] (XGPRT), P0A8F0 [https://www.uniprot.org/uniprotkb/P0A8F0/entry] (UPRT), P0A7E9 [https://www.uniprot.org/uniprotkb/P0A7E9/entry] (UMPK), P0A7E5 [https://www.uniprot.org/uniprotkb/P0A7E5/entry] (CTPS), Q04637 [https://www.uniprot.org/uniprotkb/Q04637/entry] (eIF4G), and P60842 [https://www.uniprot.org/uniprotkb/P60842/entry] (eIF4A). Source data are provided with this paper.

Human research participants

Policy information about studies involving human research participants and Sex and Gender in Research.

Reporting on sex and gender	N/A
Population characteristics	N/A
Recruitment	N/A
Ethics oversight	N/A

Note that full information on the approval of the study protocol must also be provided in the manuscript.

Field-specific reporting

Please select the one below that is the best fit for your research. If you are not sure, read the appropriate sections before making your selection.

☒ Life sciences ☐ Behavioural & social sciences ☐ Ecological, evolutionary & environmental sciences

For a reference copy of the document with all sections, see nature.com/documents/nr-reporting-summary-flat.pdf

Life sciences study design

All studies must disclose on these points even when the disclosure is negative.

Sample size	No statistical methods were used to predetermine sample size. For ITC data, at least two biological replicates were performed to confirm that the binding is not determined. For in vitro translation assay, we chose the sample size of 7 according to our own preliminary experiments. For relaxation dispersion NMR analyses, the number of points were determined from the relaxation delays with which sufficient signal-to-ratio can be achieved, determined from our own preliminary experiments. Cryo-EM data sample size was chosen based on the sufficient number of particles to obtain the high resolution constructions.
Data exclusions	The data were not excluded from the analyses.
Replication	All attempts at replication were successful. The numbers of experiments are described in the Methods section or figure legend, where applicable.
Randomization	In the Monte Carlo analysis for the relaxation dispersion fitting, data points were randomly eliminated to estimate the error. Randomization method was not utilized to the other methods because sample bias is impossible for our biochemical studies due to the use of experimental replicates.

Blinding	Blinding is not relevant for this study because no group allocation was performed.

Reporting for specific materials, systems and methods

We require information from authors about some types of materials, experimental systems and methods used in many studies. Here, indicate whether each material, system or method listed is relevant to your study. If you are not sure if a list item applies to your research, read the appropriate section before selecting a response.

Materials & experimental systems

n/a	Involved in the study
☒	☐ Antibodies
☒	☐ Eukaryotic cell lines
☒	☐ Palaeontology and archaeology
☒	☐ Animals and other organisms
☒	☐ Clinical data
☒	☐ Dual use research of concern

Methods

n/a	Involved in the study
☒	☐ ChIP-seq
☒	☐ Flow cytometry
☒	☐ MRI-based neuroimaging
